# The Effect of SARS-CoV-2 İnfection on Perinatal Outcomes in Hypertensive Disorders of Pregnancy

**DOI:** 10.1055/s-0043-1772184

**Published:** 2023-09-08

**Authors:** Nihat Farisoğulları, Ramazan Denizli, Bedri Sakcak, Atakan Tanaçan, Özgür Kara, Dilek Şahin

**Affiliations:** 1 Division of Perinatology, Department of Obstetrics and Gynecology, Turkish Ministry of Health Ankara City Hospital, Ankara, Turkey.; 2 Division of Perinatology, Department of Obstetrics and Gynecology, University of Health Sciences, Turkish Ministry of Health Ankara City Hospital, Ankara, Turkey.

**Keywords:** COVID-19, hypertensive disorders of pregnancy, perinatal outcomes, SARS-CoV-2, preeclampsia

## Abstract

**Objective** To evaluate the fetal and maternal effects of the severe acute respiratory syndrome virus 2 (SARS-CoV-2) infection in women with hypertensive disorders of pregnancy.

**Methods** Patients with hypertensive disorders of pregnancy and SARS-CoV-2 polymerase chain reaction (PCR) positivity ( *n* = 55) were compared with cases with similar characteristics and PCR negativity ( *n* = 53). The study group was further divided into two groups as severe ( *n* = 11) and nonsevere ( *n* = 44) coronavirus disease 2019 (COVID-19). The groups were compared in terms of clinical characteristics and perinatal outcomes.

**Results** The study and control groups were similar in terms of maternal age, parity, gestational age at diagnosis, type of hypertensive disorders, magnesium sulfate administration rate, gestational age at birth, birth weight, Apgar scores, and maternal complications. However, all cases of fetal loss ( *n* = 6) were observed in the SARS-CoV-2 positive group ( *p* = 0.027). From the 6 cases, there were 5 in the nonsevere group and 1 patient in the severe SARS-CoV-2 positive group. Moreover, higher rates of maternal complications, lower oxygen saturation values, and intensive care unit admissions were observed in the severe COVID-19 group.

**Conclusion** Physicians should be cautious about the management of hypertensive disorders of pregnancy cases with SARS-CoV-2 positivity. Fetal loss seems to be more common in cases with SARS-CoV-2 positivity and severe COVID-19 seems to be associated with higher rates of maternal complications. Close follow-up for fetal wellbeing and active management of severe cases in terms of maternal complications seem to be favorable.

## Introduction

The coronavirus disease 2019 (COVID-19) caused by severe acute respiratory syndrome coronavirus 2 (SARS-CoV-2) infection has been declared a pandemic by the World Health Organization (WHO) on March 11, 2020. 1 The primary effect of SARS-CoV-2 is on the lungs, followed by liver damage, thrombocytopenia, hypertension, and kidney damage. 2 The reason why the virus can cause disease in many organs is believed to be due to the angiotensin-converting enzyme-2 (ACE-2) receptor. The virus binds to the ACE-2 receptor, causing endothelial dysfunction, vasoconstriction, and increased vascular resistance. 3 It is known that SARS-CoV-2 infection directly causes endothelial damage and leads to vascular damage, together with diffuse thrombosis and microangiopathy. 4

Hypertensive disorders of pregnancy (HDP) occur in 6 to 8% of pregnant women and cause significant maternal or fetal morbidity and mortality. 5 These disorders are considered to cause the widespread activation and dysfunction of the maternal vascular endothelium, resulting in increased sensitivity to angiotensin II. 6 The increased expression of adhesion molecules on the activated endothelium intensifies the inflammation process and results in further endothelial damage. 7

During pregnancy, COVID-19 causes specific vascular pathology and inflammation similar to preeclampsia, gestational hypertension, and essential hypertension. 8 Considering this similar pathophysiology, we aimed to evaluate the fetal and maternal effects of SARS-CoV-2 infection in women with HDP. We also aimed to compare severe and nonsevere COVID-19 cases in the same patient group.

## Methods

Patients who presented to the hospital over a 2-year period from April 15, 2020, through April 15, 2022, were retrospectively analyzed. All pregnant women admitted to our hospital, regardless of symptoms, were tested for COVID-19 as a requirement of universal screening. The study group consisted of 55 patients aged 18 to 49 years, with a single pregnancy at 20 to 40 gestational weeks, who were diagnosed with HDP, tested positive for SARS-CoV-2 in the polymerase chain reaction (PCR) test, and did not have a history of COVID-19 vaccination. The control group comprised 53 patients diagnosed with HDP, who did not have COVID-19 during pregnancy, did not have a history of COVID-19 vaccination, and had a negative result in the COVID-19 PCR test. The control group was selected from cases with similar demographic features who were hospitalized for HDP and tested negative for SARS-CoV-2. We further divided the study group into two, with severe ( *n* = 11) and non-severe ( *n* = 44) cases according to the course of COVID-19. Patients with chronic liver and kidney diseases, history of malignancy, and known lung and heart diseases were excluded from the study.

Informed consent was obtained from all individuals included in this study. Approval for the study was obtained from the local ethics committee and the Turkish Ministry of Health.

The participants' demographic data, symptoms, vital signs, laboratory results, obstetric histories, pregnancy outcomes, obstetric and maternal complications, and the COVID-19 disease course were examined and recorded in the case report form. Intrauterine growth restriction, oligohydramnios, fetal tachycardia, placental abruption, and intrauterine fetal death were assessed for the obstetric complications. Maternal complications included wound infection, hemorrhage, relaparotomy requirement, hemolysis elevated liver enzymes low platelets (HELLP), pulmonary edema, intracranial complication, dialysis requirement, and death.

The diagnosis of COVID-19 was made based on a positive test result in the PCR analysis of combined nasopharyngeal and oropharyngeal swab samples. 9 Severe COVID-19 was defined as the presence of dyspnea, respiratory rate of 30 or greater per minute, oxygen saturation of 93% or less in room air, or findings consistent with pneumonia, while patients with a positive PCR test for SARS-CoV-2 without severe symptoms of the disease were evaluated to have the nonsevere disease. 10

The HDP were divided into the following four categories, according to the criteria recommended by the American College of Obstetricians and Gynecologists: preeclampsia/eclampsia, chronic hypertension, chronic hypertension with superimposed preeclampsia, and gestational hypertension. Gestational hypertension was defined as hypertension that occurred for the first time after the 20 ^th^ week of pregnancy in the absence of proteinuria and continued for 6 weeks after pregnancy. Chronic hypertension as a blood pressure equal to or higher than 140/90 mm Hg before pregnancy or before the 20 ^th^ week of pregnancy. Preeclampsia as a blood pressure of 140/90 mm Hg or greater and proteinuria or accompanied by significant end-organ dysfunction without proteinuria that developed after 20 weeks. Superimposed preeclampsia is the addition of new-onset proteinuria or other preeclampsia findings to new-onset or progressing hypertension in pregnant women with documented chronic hypertension. Finally, eclampsia is the convulsive form of preeclampsia. 11

The Statistical Package Social Sciences (SPSS, IBM Corp. Armonk, NY, USA) was used for statistical analyses. The Kolmogorov-Smirnov test was conducted to determine whether the data with continuous values fit the normal distribution. The Student *t* -test was used to evaluate normally distributed (parametric) variables between the two groups. The chi-square test was performed to compare categorical variables. The statistical significance level was set as *p* < 0.05.

## Results

During the study period, 108 patients diagnosed with HDP were included in the study. Of these patients, 55 constituted the SARS-COV-2-positive study group and 53 constituted the negative control group. The patients in the positive study group were further divided into two groups as nonsevere ( *n* = 44) and severe ( *n* = 11) COVID-19 cases.

Demographic characteristics such as age, gravida, parity, gestational age at diagnosis, and body mass index (BMI) were similar in the study and control groups. The systolic and diastolic blood pressure values at the time of hospitalization were statistically significantly higher in the control group. Concerning the laboratory data, the alanine aminotransferase (ALT) value was significantly higher in the study group, and the creatinine level was significantly higher in the control group ( *p* < 0.05). There were no significant differences between the two groups in relation to the aspartate aminotransferase (AST), platelet, and lactate dehydrogenase (LDH) values ( Table 1 ).

**Table 1:** Comparison of demographic data and laboratory results of the groups

	SARS-CoV-2 positive ( *n* = 55)	SARS-CoV-2 negative ( *n* = 53)	*p* -value
Maternal age (years)	31 ± 6.5	29.5 ± 6.1	0.313
BMI (kg/m ^2^ )	29.2 ± 4	30.3 ± 4.1	0.166
Gravida	2.9 ± 1.8	2.3 ± 1.3	0.126
Parity, n (%)			0.125
Primiparous	18 (32.7)	25 (47.2)	
Multiparous	37 (67.3)	28 (52.8)	
Hypertensive disorders, n (%)			0.244
Chronic hypertension	2 (3.6)	2 (3.8)	
Superimposed preeclampsia	4 (7.2)	6 (11.3)	
Gestational hypertension	13 (23.6)	11 (20.8)	
Preeclampsia	35 (63.6)	34 (64.2)	
Eclampsia	1 (1.8)	0	
Gestational age at diagnosis (weeks)	32.1 ± 4.7	33.3 ± 3.7	0.144
Systolic blood pressure (mmHg)*	138.9 ± 25	149.7 ± 13.4	0.002
Diastolic blood pressure (mmHg)*	82.1 ± 14	94.6 ± 8.7	<0.001
AST (U/L)	45.4 ± 52.6	22.2 ± 15.5	0.080
ALT (U/L)	42.2 ± 54.9	16.8 ± 11.9	0.023
Platelets/µL	235.4 ± 96.3	233.9 ± 60.5	0.907
LDH (U/L)	303 ± 107.8	266.2 ± 87.1	0.098
Creatinine (mg/dL)	0.6 ± 0.3	0.6 ± 0.1	0.036

**Abbreviations:** AST, aspartate aminotransferase; ALT, alanine aminotransferase; BMI, body mass index; LDH, lactate dehydrogenase; SARS-CoV-2, severe acute respiratory syndrome coronavirus 2. **Notes:** Values are expressed as mean ± standard deviation or number (percentage). *The blood pressures of the patients were measured during hospitalization. Other laboratory parameters are the values at the time of admission to the hospital.

The oxygen saturation was higher in the study group than in the control group. The two groups were statistically similar in terms of delivery method, newborn weight, APGAR 1 ^st^ - and 5 ^th^ -minute scores, length of hospital stay, intensive care requirement.

The rate of maternal complications was 20% (11/55) in the study group and 9.4% (5/53) in the control group, with no statistically significant difference. Wound infection was the most common maternal complication in both groups.

Obstetric complications were found at a statistically similar rate—27.2% (15/55) in the study group and 22.6% (12/53) in the control group. When the obstetric complications were examined individually, intrauterine fetal death (IFD) was observed in 6 patients in the study group and no patient in the control group, which was a statistically significant difference ( *p* = 0.027) ( Table 2 ).

**Table 2:** Comparison of obstetric complications, maternal complications, and perinatal outcomes between the groups

	SARS-CoV-2 positive ( *n* = 55)	SARS-CoV-2 negative ( *n* = 53)	*p* -value
Delivery week, n (%)			0.217
<34 weeks	14 (25.5)	9 (17.0)	
34–37 weeks	21 (38.2)	16 (30.2)	
>37 weeks	20 (36.4)	28 (52.8)	
Delivery method, n (%)			0.616
Cesarean section	44 (80)	45 (84.9)	
Vaginal delivery	11 (20)	8 (15.1)	
Newborn weight (g)	2,304.8 ± 848.2	2,553.9 ± 824.3	0.103
1-minute Apgar score <7, n (%)	11 (22.4)	14 (26.4)	0.642
5-minute Apgar score <7, n (%)	5 (10.2)	3 (5.7)	0.394
Admission to neonatal intensive care unit, n (%)	14 (28.6)	19 (35.8)	0.432
Hospitalization (day)	5.1 ± 4.8	3.6 ± 1.7	0.216
Admission to intensive care unit, n (%)	7 (12.7)	3 (5.7)	0.205
Oxygen saturation	95.1 ± 2.6	96.3 ± 1.2	0.010
Magnesium sulfate therapy, n (%)			0.482
Yes	22 (40)	25 (47.2)	
No	33 (60)	28 (52.8)	
Maternal complications, n (%)			0.632
Wound infection	4 (7.3)	2 (3.8)	
Hemorrhage	0	1 (1.9)	
Relaparotomy requirement	1 (1.8)	1 (1.9)	
HELLP	2 (3.6)	1 (1.9)	
Pulmonary edema	1 (1.8)	0	
Intracranial complication	1 (1.8)	0	
Dialysis requirement ^a^	1 (1.8)	0	
Death	1 (1.8)	0	
Obstetric complications, n (%)			0.250
Intrauterine growth restriction	3 (5.5)	4 (7.6)	
Oligohydramnios	3 (5.5)	5 (9.4)	
Fetal tachycardia	1 (1.8)	1 (1.9)	
Placental abruption	2 (3.6)	2 (3.8)	
Intrauterine fetal death ^b^	6 (10.9)	0	0.027

**Abbreviation:** HELLP, Hemolysis elevated liver enzymes low platelets; SARS-CoV-2, severe acute respiratory syndrome coronavirus 2. **Notes:** ^a^ Lupus nephritis was detected as a result of examinations and kidney biopsy (diffuse proliferative glomeruloneritis). The patient started to receive dialysis for the first time in the postpartum period. ^b^ When the obstetric complications were examined individually, a difference was observed between the groups in terms of the number of intrauterine fetal deaths. Values are expressed as mean ± standard deviation or number (percentage).

There were 6 cases of IFD between 24 and 33 weeks. The weeks of cases are 24 (2 cases), 25, 29, 30, and 33 weeks. Intrauterine fetal death was observed 2 to 14 days after SARS-CoV-2 positivity (2,3,4,7,9, and 14 days). Placental abruption was observed in one patient. Furthermore, one of them was found to have intrauterine growth restriction. An IFD case was found to have severe SARS-CoV-2 and preterm premature rupture of membranes (PPROM). In another, her blood pressure was 160/110 mm Hg at the time of admission to the hospital, and she was diagnosed with severe preeclampsia. One case was diagnosed with HELLP. In one patient, no additional pathology was observed other than COVID-19 and gestational hypertension. Thus, at least one severe obstetric complication was present in 5 of the 6 cases with IFD. Placental specimen evaluation was performed in all cases. The main findings were placental infarction, intervillous hemorrhage, focal necrosis, and increased inflammation.

When we divided the study group into two subgroups according to the severity of the disease (severe and nonsevere COVID-19), we determined that the demographic data and laboratory results of these groups were similar ( Table 3 ).

**Table 3:** Comparison of the demographic data and laboratory results of the study group according to the severity of the disease

	Non-severe SARS-CoV-2 ( *n* = 44)	Severe SARS-CoV-2 ( *n* = 11)	*p* -value
Maternal age (years)	31.2 ± 6	30.5 ± 8.5	0.643
BMI (kg/m ^2^ )	29.2 ± 4.1	29.1 ± 3.7	0.929
Gravida	2.9 ± 1.9	2.6 ± 1.6	0.763
Parity, n (%)			0.774
Primiparous	14 (31.8)	4 (36.4)	
Multiparous	30 (68.2)	7 (63.6)	
Hypertensive disorders, n (%)			0.101
Chronic hypertension	2 (4.5)	1 (9.1)	
Superimposed preeclampsia	3 (6.8)	0	
Gestational hypertension	12 (27.3)	1 (9.1)	
Preeclampsia	27 (61.4)	8 (72.7)	
Eclampsia	0	1 (9.1)	
Gestational age at diagnosis (week)	32 ± 4.6	32.5 ± 5.4	0.598
Systolic blood pressure (mmHg)*	140.9 ± 24.6	130.9 ± 25.7	0.335
Diastolic blood pressure (mmHg)*	82 ± 14.1	82.5 ± 14.6	0.940
AST (U/L)	45.8 ± 55.7	43.6 ± 40.5	0.792
ALT (U/L)	43.1 ± 59.7	38.2 ± 30.6	0.406
Platelets/µL	226.4 ± 94.6	271.6 ± 99.2	0.230
LDH (U/L)	299.7 ± 107.3	316 ± 114.1	0.570
Creatinine (mg/dL)	0.5 ± 0.2	0.7 ± 0.5	0.643

**Abbreviations:** AST, aspartate aminotransferase; ALT, alanine aminotransferase; BMI, body mass index; LDH, lactate dehydrogenase; SARS-CoV-2, severe acute respiratory syndrome coronavirus 2. **Notes:** Values are expressed as mean ± standard deviation or number (percentage). *The blood pressure of the patients was measured during hospitalization. Other laboratory parameters are the values at the time of admission to the hospital.

Initial symptoms at hospitalization were similar in both groups. Perinatal outcomes were similar between these two groups. While intensive care requirement was higher in those with severe disease, the saturation value was lower. The rates of antihypertensive use and magnesium prophylaxis treatment were similar between the two groups. Hospital stay was longer in the severe group ( *p* = 0.033). Maternal complications were seen in 15.9% (7/44) of the patients in the nonsevere group and in 36.3% (4/11) of those in the severe group, indicating a statistically significant difference ( *p* = 0.011). Obstetric complications occurred at a rate of 29.5% (13/44) in the nonsevere group and 18.1% (2/11) in the severe group, which was not statistically significant ( Table 4 ).

**Table 4:** Comparison of the obstetric complications, maternal complications, and perinatal outcomes of the study group according to the severity of the disease

	Non-severe SARS-CoV-2 ( *n* = 44)	Severe SARS-CoV-2 ( *n* = 11)	*p* -value
Delivery week, n (%)			0.617
<34 weeks	10 (22.7)	4 (36.4)	
34–37 weeks	17 (38.6)	4 (36.4)	
>37 weeks	17 (38.6)	3 (27.3)	
Delivery method, n (%)			0.866
Cesarean section	35 (79.5)	9 (81.8)	
Vaginal delivery	9 (20.5)	2 (18.2)	
Newborn weight (g)	2,320.9 ± 875.1	2,240.5 ± 766	0.760
1-minute Apgar score <7, n (%)	8 (20.5)	3 (30)	0.521
5-minute Apgar score <7, n (%)	4 (10.3)	1 (10)	0.981
Admission to neonatal intensive care unit, n (%)	11 (28.2)	3 (30)	0.911
Hospitalization (day)	3.8 ± 1.6	10.3 ± 8.7	0.033
Admission to intensive care unit, n (%)	2 (4.5)	5 (45.5)	<0.001
Oxygen saturation	96.1 ± 0.9	90.7 ± 2.6	<0.001
Initial symptoms, n (%)			0.106
Asymptomatic	24 (54.5)	3 (27.3)	
Symptomatic	20 (45.5)	8 (72.7)	
Cough	6 (13.6)	3 (27.3)	
Dyspnea	7 (15.9)	1 (9.1)	
Fever	2 (4.6)	1 (9.1)	
Headache	4 (9.1)	1 (9.1)	
Sore throat	1 (2.3)	1 (9.1)	
Chest pain	0	1 (9.1)	
Antihypertensive therapy, n (%)			0.291
Yes	16 (36.4)	5 (45.5)	
No	28 (63.6)	6 (54.5)	
Magnesium sulfate therapy, n (%)			0.783
Yes	18 (40.9)	4 (36.4)	
No	26 (59.1)	7 (63.6)	
Maternal complications, n (%)			0.011
Wound infection	4 (9)	0	
Hemorrhage	0	0	
Relaparotomy requirement	1 (2.3)	0	
HELLP	2 (4.6)	0	
Pulmonary edema	0	1 (9)	
Intracranial complication	0	1 (9)	
Dialysis requirement	0	1 (9)	
Maternal death	0	1 (9)	
Obstetric complications (n)			0.845
Intrauterine growth restriction	3 (6.8)	0	
Oligohydramnios	2 (4.6)	1 (9)	
Fetal tachycardia	1 (2.3)	0	
Placental abruption	2 (4.6)	0	
Intrauterine fetal death	5 (11.4)	1 (9)	

**Abbreviation:** HELLP, hemolysis elevated liver enzymes low platelets; SARS-CoV-2, severe acute respiratory syndrome coronavirus 2. **Notes:** Values are expressed as mean ± standard deviation or number (percentage).

## Discussion

In this study, we compared the SARS-CoV-2-positive (study group) and negative (control) patients with HDP. The study and control groups were similar in terms of perinatal outcomes, maternal complications, and obstetric complications. We further divided the study group in two subgroups according to the course of the disease as severe and nonsevere. These two subgroups were similar in terms of perinatal outcomes and obstetric complications, but maternal complications were more common in the severe COVID-19 group.

It is important to point out that HDP is an umbrella term covering a series of conditions, namely preeclampsia/eclampsia, gestational hypertension, chronic hypertension, and chronic hypertension with superimposed preeclampsia. 5 Maternal and fetal complications increase in pregnant women with HDP. Premature birth (spontaneous/iatrogenic), intrauterine growth restriction, placental abruption, and stillbirth are the most common fetal complications of HDP. 12 Intracranial complications, pulmonary edema, postpartum hemorrhage, acute renal failure, and maternal death can be listed as maternal complications. 13 Approximately 25% of maternal deaths in the United States of America have been associated with HDP. 14

Furthermore, it is known that SARS-CoV-2 infection increases the risk of preeclampsia, stillbirth, preterm birth, and neonatal intensive care requirement, and severe COVID-19 during pregnancy is a risk factor for unfavorable maternal, fetal, and neonatal outcomes. 15 It has been shown that COVID-19 during pregnancy increases severe maternal morbidity and mortality, especially respiratory dysfunction requiring invasive mechanical ventilation or admission to the intensive care unit. 16

The combination of nasopharyngeal and oropharyngeal swab that we use in every pregnant woman hospitalized in our hospital is one of the most frequently used test samples for the diagnosis of COVID-19. 17 According to the results of a report including a metanalysis, 43.5 to 92% of cases were asymptomatic at hospital admission. Fever was the most common clinical findings reported in the infected pregnant women. In one study, fever was observed less frequently in pregnant women than in nonpregnant women. 18 While fever was 5.6% in our study, the most common symptoms were cough and dyspnea, and asymptomatic patients were 49.1%. When we examined the gestational week at the time of diagnosis, 30.6 ± 9.5 weeks were observed in the WAPM study. 19 In our study, it was observed as 32.1 ± 4.7.

Studies have shown that COVID-19 modulates the expression of placental ACE-2, which may be related to the development of HDP. 20 This receptor is a new component of the renin-angiotensin aldosterone mechanism (RAAM) identified in 2000. 21 The infection with SARS-CoV-2 exhibits its effects through the ACE-2 receptor, causing vasoconstriction resulting from renin-angiotensin system dysfunction. 22 In mothers with gestational hypertension and preeclampsia, there is a marked progressive dysfunction in RAAM activity starting from early placentation. 23 The systematic endothelial dysfunction caused by HDP may share a common pathway with SARS-CoV-2 infection. 15

In the current study, in which we examined the effects of SARS-CoV-2 positivity on maternal, fetal, and obstetric outcomes in patients with HDP, we found similar results between the two groups. Although the obstetric complications were similar, when each complication was analyzed separately, we determined that the rate of IFD cases was significantly higher in the SARS-CoV-2-positive group, which may have resulted from sudden hypertensive attacks caused by the placental vascular malperfusion and ACE2/RAAM dysfunction observed in SARS-CoV-2 positive patients. 24 25

Previous studies suggest that maternal and fetal complications are more common in severe SARS-CoV-2 cases. 25 26 In one of these studies, while the rate of preterm delivery was 31.9% in severe cases, it was 13.1% in the nonsevere group. 26 In our study, the rate of preterm birth was over 70% in the severe COVID-19 group, which was statistically similar to the rate detected in the nonsevere group. The reason for the higher rate of preterm birth in our sample was due to our patients being also hypertensive. In a metanalysis, it was shown that the rate of admission to the neonatal intensive care unit was higher in severe COVID-19 cases. 4 In our study, we did not observe a significant difference between the severe and nonsevere COVID-19 group in relation to neonatal intensive care requirement.

The two main mechanisms implicated in the pathophysiology of HDP are placental dysfunction and systemic inflammation. 6 Similarly, SARS-CoV-2 infection not only impairs placental function but also causes an uncontrolled inflammatory response in the host through increased inflammation and thrombotic tendency mechanisms. 3 Thus, the clinical course and pregnancy outcomes of hypertensive SARS-CoV-2 positive cases remain a matter of curiosity. Currently, there are no adequate and satisfactory studies on this subject in the literature. Therefore, the current study is one of the first in this area and has the potential to guide further similar studies that can be conducted in future. However, we found no significant difference between the groups regarding the majority of perinatal outcomes. This can be attributed to the relatively small sample size and the limited number of severe COVID-19 cases.

The most significant findings obtained from the present study were the higher rate of IFD cases in the SARS-CoV-2 positive group. Additionally, nearly all cases had at least one accompanying severe obstetric complication and placental pathologic assessment indicated vascular pathologies together with increased inflammation. In our opinion, the potential adverse impact of SARS-CoV-2 on the growing conception material might lead to IFD. However, more data are necessary to confirm these results. Furthermore, the increased rate of maternal complications in the severe COVID-19 group was also an important finding. As HDP was associated with vascular dysfunction and increased inflammation, additional infectious agents like SARS-CoV-2 might lead to adverse outcomes. Thus, optimal care should be provided in the management of these complicated cases.

The strengths of the study are the multiplicity of investigated parameters and its unique design. However, it also had certain limitations, including its retrospective design and the relatively low number of patients in the sample.

## Conclusion

Physicians should be cautious about the management of hypertensive disorders of pregnancy cases with SARS-CoV-2 positivity. Close follow-up for fetal wellbeing and active management of severe cases in terms of maternal complications seem to be favorable.
